# A Rare Case of Rapid Lymphadenopathy Progression in Hodgkin’s Lymphoma: A Case Report

**DOI:** 10.7759/cureus.57954

**Published:** 2024-04-10

**Authors:** Harsh Bhalala, Yacoub Faroun, Wenxin Liao

**Affiliations:** 1 Internal Medicine, St. Luke's University Health Network, Bethlehem, USA; 2 Department of Oncology, St. Luke’s University Hospital–Bethlehem Campus, Bethlehem, USA

**Keywords:** reed-sternberg cells, non hodgkin's lymphoma, hematologic malignancy, hodgkin's lymphoma, rapid onset lymphadenopathy

## Abstract

Hodgkin’s lymphoma is a B-cell neoplasm that typically manifests with gradual lymphadenopathy progression over weeks to months. However, we present an exceptional case of Hodgkin’s lymphoma marked by an unusually rapid development of lymphadenopathy within an hour. A 30-year-old male presented with a left neck swelling that occurred within an hour and then remained stable in size for three days, prompting an investigation revealing widespread lymphadenopathy consistent with Hodgkin’s lymphoma. This case outlines the importance of recognizing and investigating unusual presentations of Hodgkin’s lymphoma promptly, emphasizing the necessity for expedited diagnosis and intervention.

## Introduction

Hodgkin lymphoma (HL) is a B-lymphocytic neoplasm originating in the lymphatic system, identified on pathology by characteristic malignant Hodgkin and Reed-Sternberg cells (HRS) interspersed with a multitude of inflammatory cells [1]. The estimated incidence rate in the United States is around 2.6 cases per 100,000 as of 2023, with a bimodal distribution containing a majority of cases from ages 20-40 and another peak from age 55 and older [2]. The typical clinical presentation begins with gradually progressive painless lymphadenopathy in the neck (60% of cases) or mediastinum (20% of cases). Other common symptoms include night sweats, fevers, and weight loss [3]. Here, we discuss a case of HL in a patient who presented with a very rapid expansion of cervical lymph nodes without other symptoms.

## Case presentation

A 30-year-old male with no significant past medical history presented with acute left neck swelling that began three days ago while he was camping. The growth occurred within an hour and remained stable in size afterward. The patient's family members corroborated the same. He reported no swelling elsewhere. He did notice that his dog had ticks, but he did not find any insect bites. He had not sought any other treatment at that time. He described no symptoms of fever, night sweats, or recent weight loss. He reported no dyspnea, dysphagia, or dysarthria. He did not report any contact with people with similar symptoms or any recent traumatic events.

**Figure 1:**
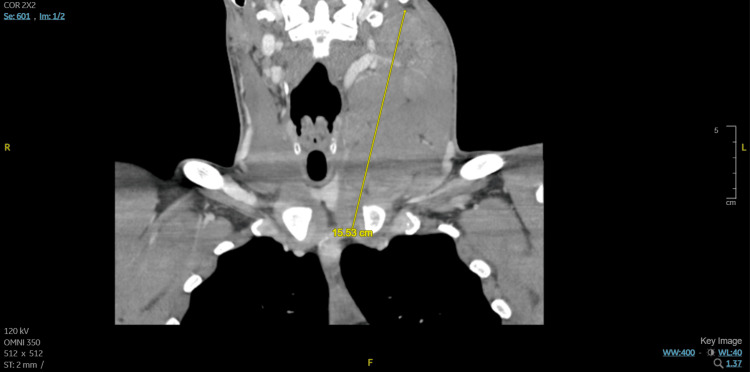
Computed tomography soft tissue neck with contrast with yellow line demonstrating enlarged left cervical lymph node conglomerate measuring 15.5 cm with significant mass effect

The physical examination revealed an otherwise fit-looking young man with a left-sided mass noted in the anterior cervical region. The range of motion of the neck was full and painless, with no tenderness on palpation. He received doxycycline 100 mg orally due to suspicion of Lyme disease. Relevant workups included HIV (Human Immunodeficiency Virus), Lyme serology, which returned negative, and cultures that found no growth. Tuberculosis testing was not performed. No inflammatory markers were checked. Computed tomography (CT) soft tissue neck with contrast (Figure 1) revealed an enlarged left cervical lymph node conglomerate measuring 15.5 cm with significant mass effect, with CT chest abdomen pelvis finding mild upper mediastinal, aortocaval, and external iliac lymphadenopathy.

Due to suspicion of a potential malignant process, further workup was pursued. An interventional radiology-guided core needle biopsy of the left cervical lymph node was obtained. Flow cytometry of the left cervical lymph node biopsy specimen showed no evidence of B or T-cell lymphoma. However, a histological tissue exam showed a background of mixed inflammatory cells, including scattered plasma cells, abundant eosinophils, abundant histiocytes, and both small and mature lymphocytes with scattered Hodgkin cells and Reed-Sternberg cells, confirming the to be classic HL likely mixed cellularity subtype. By immunohistochemistry, the large cells were positive for CD30, PAX5 (dim), CD15 (subset), MUM-1, OCT-2 (subset dim), BOB.1 (subset dim), fascin, BCL1 (subset dim), p53 (subset dim), EBV-encoded RNA in situ hybridization (EBER ISH), and EBV (LMP1). PET (positron emission tomography)-CT further confirmed lymphoma with bulky left cervical lymphadenopathy, avid osseous lesions, and heterogeneous foci in the spleen with Deauville classification 5. Cytogenetic fluorescence in situ hybridization (FISH) analysis was completed and found negative for BCL2, BCL6, MYC, and IRF4. Eventually, the patient elected to be treated at a different institute, and hence further evaluation, treatment, and outcome data are not available.

## Discussion

The etiology of HL is not fully understood. There is a known association with the Epstein-Barr virus (EBV). EBV is often found in HRS cells, particularly in HL patients from other countries outside the United States and Europe [4]. The risk of developing EBV-positive HL is greatly increased (relative risk, 4.0; 95 percent confidence interval, 3.4 to 4.5) after EBV infectious mononucleosis, but the absolute risk is only noted to be around one in 1000 [4,5].

Immunosuppression is also associated with an increased risk of HL, such as in patients receiving organ transplants or in those who are HIV-infected. For such higher-risk individuals, EBV is found in up to 90% of HL occurring in the first year after transplant or people living with HIV. There is evidence to suggest that people living with HIV have a 10-fold greater risk of HL than those without HIV [1,5]. Autoimmune disorders such as rheumatoid arthritis, systemic lupus erythematosus, and Sjögren’s syndrome are also associated with an increased incidence of HL, with an overall odds ratio for systemic autoimmune disorders being 2.7-fold (1.9-3.8) elevated [1,6].

The WHO classification of classic HL (cHL) is subdivided into four histologic subtypes: lymphocyte-rich (LRCHL), lymphocyte-depleted (LDCHL), mixed cellularity (MCCHL), and nodular sclerosis (NSCHL). LRCHL presents with isolated lymphadenopathy without B symptoms; LDCHL presents with B symptoms and a more aggressive spread in elderly adults; MCCHL presents with peripheral lymphadenopathy in children or elderly adults; and NSCHL often presents with mediastinal mass (80% patients) in adolescents or young adults [7,8].

On histology, NSCHL shows HRS cells with encircling, thick collagen banding. Both MCCHL and LDCHL show HRS cells with varied surrounding leukocytes, such as neutrophils and eosinophils. LRCHL shows a background consisting of a majority of B-lymphocytes that form nodules [7].

The staging of HL follows the Lugano classification (Table 1) and is subdivided into early favorable (stages I-II), early unfavorable, and advanced stages.

**Table 1:** Lugano staging classification for lymphoma [8]

Stage	Description	Involvement	Systemic Symptoms
I	Single node or group of adjacent nodes	Nodal or extranodal	A (absence) or B (presence)
IE	Single extra-lymphatic site without nodal involvement	Extranodal	A or B
II	Two or more nodal groups on the same side of the diaphragm	Nodal	A or B
IIE	Contiguous extra-lymphatic extension from a nodal site	Extranodal	A or B
III	Nodes on both sides of the diaphragm	Nodal	A or B
III (1)	Involvement of spleen or splenic, hilar, celiac, or portal nodes	Nodal	A or B
III (2)	Involvement of para-aortic, iliac, inguinal, or mesenteric nodes	Nodal	A or B
IV	Diffuse or disseminated involvement of extranodal organs or tissues	Extranodal	A or B

The formal definition for B symptoms is a fever with a temperature > 38°C (> 100.4°F), the presence of drenching night sweats, and an unexplained loss of > 10 percent body weight in the last six months [8]. These are included in the staging classification, with A indicating asymptomatic and B indicating the presence of such symptoms. Bulky disease is defined as a lymph node mass > 10 cm or a mediastinal mass taking up over a third of the transthoracic diameter on CT.

Prognostic factors for unfavorable outcomes as per the German Hodgkin Study Group (GHSG) for early-stage HL include the following: one or more risk factors present; three or more lymph node involvement; erythrocyte sedimentation rate (ESR) >/= 50; mediastinal mass ratio (MMR) > 1/3; the presence of extranodal disease [9,10]. Those for late stage are the following: serum albumin < 4 g/dL, hemoglobin < 10.5 g/dL, male sex, stage IV disease, age > 45, leukocyte count > 15,000/mm3, and lymphocyte count < 600/mm3, or < 8% of the white-cell count [11].

Treatment for early-stage HL is a combination of shorter-course chemotherapy with involved-field radiation therapy. Positron emission tomography/computed tomography (PET/CT) is used to functionally assess the response to initial chemotherapy (e.g., doxorubicin (adriamycin), bleomycin, vinblastine, and dacarbazine (ABVD)), guide the duration of chemotherapy (i.e., the number of chemotherapy cycles), and determine if any residual disease might be encompassed within a radiation field. Treatment for advanced-stage HL involves longer courses of chemotherapy, with radiation being only an additional option. ABVD chemotherapy is the most common choice, being a combination of doxorubicin, bleomycin, vinblastine, and dacarbazine. Overall, there is a favorable prognosis with cure rates as high as > 90%, so the long-term risk of such therapies is taken into account when formulating treatments. However, the risk of relapse or failure to respond to treatment remains a challenge [1,10,12].

## Conclusions

Classic Hodgkin lymphoma is a cancer that disproportionately affects both young and older people. Our young patient displayed a rapid onset of isolated lymphadenopathy within the span of a few hours, with swift progression that has not been reported in any prior case studies. Nowadays, we have a much greater understanding of the different types of Hodgkin’s lymphoma due to various histologic tests for more specificity in diagnosis. Great strides have been made in treatment regimens for Hodgkin lymphoma, with cure rates up to 90% due to tailoring of treatment to prognostic factors to control for unwanted toxicity; hence, it is of prime importance to have an eye of suspicion for a malignant process even with such an acute presentation of lymphadenopathy to prevent any delay in diagnosis and treatment of a nearly curable malignancy. 
